# Wide-field three-photon excitation in biological samples

**DOI:** 10.1038/lsa.2016.255

**Published:** 2017-05-05

**Authors:** Christopher J Rowlands, Demian Park, Oliver T Bruns, Kiryl D Piatkevich, Dai Fukumura, Rakesh K Jain, Moungi G Bawendi, Edward S Boyden, Peter TC So

**Affiliations:** 1 Department of Biological Engineering, Massachusetts Institute of Technology, Cambridge, MA 02139, USA; 2 Media Lab, Massachusetts Institute of Technology, Cambridge, MA 02139, USA; 3 Department of Chemistry, Massachusetts Institute of Technology, Cambridge, MA 02139, USA; 4 Edwin L. Steele Laboratories, Department of Radiation Oncology, Massachusetts General Hospital and Harvard Medical School, Boston, MA 02114, USA; 5 Department of Brain and Cognitive Sciences, McGovern Institute and MIT Center for Neurobiological Engineering, Massachusetts Institute of Technology, Cambridge, MA 02139-4307, USA; 6 Department of Mechanical Engineering, Massachusetts Institute of Technology, Cambridge, MA 02139, USA

**Keywords:** biophotonics, multiphoton microscopy, optogenetics, temporal focusing, three-photon

## Abstract

Three-photon wide-field depth-resolved excitation is used to overcome some of the limitations in conventional point-scanning two- and three-photon microscopy. Excitation of chromophores as diverse as channelrhodopsins and quantum dots is shown, and a penetration depth of more than 700 μm into fixed scattering brain tissue is achieved, approximately twice as deep as that achieved using two-photon wide-field excitation. Compatibility with live animal experiments is confirmed by imaging the cerebral vasculature of an anesthetized mouse; a complete focal stack was obtained without any evidence of photodamage. As an additional validation of the utility of wide-field three-photon excitation, functional excitation is demonstrated by performing three-photon optogenetic stimulation of cultured mouse hippocampal neurons expressing a channelrhodopsin; action potentials could reliably be excited without causing photodamage.

## Introduction

While two-photon microscopy is currently the method of choice for exciting chromophores with close-to diffraction-limited resolution hundreds of microns into biological tissue^1^, three-photon has several advantages over conventional two-photon excitation. The longer wavelengths used in three-photon microscopy undergo less scattering^2^; also the cubic power dependence serves to reduce out-of-focus excitation. This excitation is responsible for reducing contrast, and ultimately limits the achievable penetration depth in multiphoton microscopy^3^. Disadvantages of three-photon excitation include increased absorption at longer infrared wavelengths^2^, the need for optical pulses with a higher peak power compared with two-photon microscopy and, on occasion, the requirement for custom-built light sources^3^.

These disadvantages have limited the use of three-photon excitation to point-scanning of an excitation spot^3, 4^, despite the fact that point-scanning is either suboptimal or unsuitable for several optical techniques, such as optogenetic excitation (where it is necessary to excite as many opsins as possible in a short space of time)^5^, high-throughput imaging^6^ (where being able to image many spots in parallel is one means of increasing image throughput), stroboscopic imaging of fast processes^7^ (where all points in the image must be illuminated simultaneously), and targeted photodynamic therapy^8^ (in which pixel dwell times are necessarily long, and hence point-scanning is an inefficient use of time), to name but a few.

In this paper, we report the use of commercially available light sources to perform depth-resolved wide-field three-photon excitation of chromophores in fixed and live biological samples, with an excitation wavelength of 1300 nm. Although this type of instrument cannot image at depths approaching that of point-scanning multiphoton microscopy, due to scattering of the emission photons^9^, in applications such as photodynamic therapy or optogenetic excitation, penetration depths compete with, or potentially even exceed^10, 11^, those achievable by point-scanning.

Since this is the first example of wide-field three-photon excitation, we first image quantum dots (QDs), demonstrating that they can be excited in fixed biological samples, followed by *in vivo* imaging of the cerebral vasculature in a mouse. To demonstrate biological utility, we perform three-photon excitation of a channelrhodopsin, a class of light-sensitive ion channels each containing a retinal chromophore. These are known to be difficult to excite even under two-photon conditions^12^, owing to the need to simultaneously excite many chromophores over the surface of a cell.

## Materials and methods

### Sample preparation

QD samples were prepared by dispersing a 4 μL drop of QDs suspended in water (525 nm emitting nanocrystals provided by QD Vision) on a microscope slide. A drop of UV-curing adhesive (Norland Products NOA 74) was placed on the dried QDs, a coverslip placed on top, and the sample cured by exposure to a UV source.

HeLa cells on a glass coverslip were fixed at room temperature using 4% paraformaldehyde (Electron Microscopy Sciences 15742-10) for 10 min, permeablized for 2 × 15 min using 0.25% Triton X-100 (Sigma Aldrich 93427) in phosphate-buffered saline, and then treated with an endogenous biotin blocker (Life Technologies E21390). This was followed by treatment with Biotin-XX Phalloidin (Life Technologies B7474) for 20 min, then a 605-nm-emission QD-streptavidin conjugate (Life Technologies Q10151MP) before sealing with a coverslip using Mount Quick medium (Electron Microscopy Sciences 18000).

### Mouse vasculature imaging

Intravital images were taken through a cranial window implanted into a male nude mouse^13, 14^. The mouse was anesthetized by intraperitoneal injection of ketamine and xylazine. A tail vein catheter was placed for injecting the QD solution during imaging, however the injection was not performed until the mouse had been secured on the instrument.

The QD solution consisted of green-emitting QDs in polyethylene glycol (PEG)-phospholipid micelles, synthesized as follows: QDs were transferred into aqueous buffers using a previously reported procedure^15, 16^. Three milligrams (dry weight) QDs were mixed with 25 mg 18:1 PEG2000 PE (1,2-dioleoyl-sn-glycero-3-phosphoethanolamine-*N*-methoxy(polyethylene glycol)-2000; ammonium salt; Avanti Polar Lipids; cat. no. 880130) in chloroform. After sonication for 10 s, the solvent was removed under nitrogen flow and 2 mL isotonic saline or water was added. To completely solubilize the QDs, the aqueous solution was sonicated with a probe sonicator for 5 min, and filtered through a 0.2 μm filter. A total volume of 200 μL of this solution was injected during imaging.

Animal experiments were conducted in accordance with approved institutional protocols of MGH and MIT.

### Temporal focusing

A regenerative amplifier (Coherent Legend Elite) produced 130 fs pulses at 10 kHz, with 600 μJ pulse energy and 800 nm center wavelength. 3.2 W average power was used to pump an optical parametric amplifier (OPA; Coherent, Opera Solo with second harmonic option) that converted the 800 nm pulses to 1300 nm, with average power up to 450 mW (up to 14% conversion efficiency). After an upgrade, 5.5 W average power was used to pump the OPA, yielding up to 960 mW at 1300 nm (17% conversion efficiency).

The light from the OPA was attenuated either by altering the regenerative amplifier’s pulse compressor, by using a half waveplate (Thor Labs AHWP05M-1600) and polarizer (Newport 10GL08AR.18), or by placing a large tilted coverslip in the beam. The light then passed through a shutter (Vincent Associates VS25S2ZM1R3, suspended from an overhead gantry to avoid vibrational coupling to the table) and an 850 nm long-pass filter (Thor Labs FELH0850) to a variable beam expander (capable of × 1, × 2.5, × 3.5 and × 5 beam expansion). It then reflected off a silver mirror (Thor Labs PFSQ20-03-P01) onto a diffraction grating (Custom, Spectrogon 715.706.410 G 0750 NIR). The -1 order from the grating was imaged onto the microscope image plane through a × 0.25 telescope consisting of a 200-mm focal length, 75 mm diameter lens (Edmund Optics 86-923) and a 50 mm focal length compound lens made from two 100 mm focal length 30 mm diameter lenses (Edmund Optics 67-572). The silver mirror was placed as close to the 75 mm lens as possible, to operate the grating in a near-Littrow condition for efficiency.

After the telescope, excitation light entering the microscope was collimated by a tube lens (Zeiss 425308-0000-000), reflected off a 1200 nm short-pass dichroic mirror (Edmund Optics 86-699) and imaged onto the sample through the × 25 1.0 NA near-IR microscope objective (Olympus XLPN25XSVMP) mounted on a 400-μm travel focusing drive (Piezosystem Jena MIPOS 500 SG). The median axial full-width half-maximum (FWHM) was found to be ~3.0 μm (Supplementary Fig. 2).

The two-photon excitation path was very similar; light from the regenerative amplifier was expanded using a × 5 Galilean cylindrical telescope constructed from a -15 mm focal length lens (Thor Labs, LK1006L1-B) and 75 mm focal length lens (Edmund Optics, 69-762). The beam struck a grating (Richardson Gratings, 53006BK02-540R) at an angle of 74°, such that the -1 order diffracted beam propagated along the microscope’s optical axis. The beam was demagnified using a × 1.75 telescope before being imaged onto the intermediate image plane as before; this telescope consisted of a 3″ diameter 190 mm focal length compound lens made from two singlets (Edmund Optics 86-920 and Newport KPX232AR.16), and the same 30 mm diameter 50 mm focal length lens used for three-photon excitation. Other than a change of filter cube to a 750 nm dichroic (Semrock, FF750-SDi02-25 × 36) and 775 nm short-pass emission filter (Semrock, FF01-775/SP-25), the microscope was identical to the three-photon configuration. The median axial FWHM was found to be ~18 μm (Supplementary Fig. 3).

For imaging experiments, fluorescence emission passed through the objective, dichroic, emission filter (1200 nm short pass, Edmund Optics 86-693) and microscope tube lens, before being detected on an EMCCD camera (Andor iXon 885k). The optical layout can be seen in Supplementary Fig. 1. For the HeLa cells, the imaging parameters were 10 s integration time, × 100 EM gain on the EMCCD, 80 mW incident power and × 5 beam expander. Flat-field correction was applied to reduce the effect of non-uniform excitation.

To determine the power scaling, pure QD samples were imaged with varying excitation intensities (controlled by a tilted coverslip to minimize dispersion). Images were captured on the camera with a 1 s integration time for the 525 nm QDs, and 0.1 s for the 605 nm QDs. The excitation region was cropped, the background subtracted and the average fluorescence intensity plotted as a function of incident power.

For electrophysiology experiments, a Faraday cage was built around microscope, and an illumination system constructed, consisting of a red (Thor Labs M660L3, 660 nm center wavelength) and blue (Thor Labs M470L2, 470 nm center wavelength) LED combined using a dichroic mirror (make and model unknown; taken from a Green Fluorescent Protein filter cube) and focused onto the sample using a microscope objective (Olympus PlanN × 10/0.25). The red LED was used to locate the cells by bright-field illumination without triggering an action potential, and the blue LED was used to both excite fluorescence (in order to locate cells with high expression of CoChR) and to excite an action potential when necessary. Incident power required to excite an action potential was found to be between ~0.13 and 1.8 mW mm^−2^. For experiments establishing the maximum achievable frequency due to one-photon absorption, the intensity was optimized until the highest frequency could be achieved; these are the data reported.

To investigate the primary damage mechanism, the focal plane was placed ~180 μm from the cell surface (equal to one turn of the coarse focus knob on the microscope). Maximum laser power was used to ensure operation well above the damage threshold; this power averaged 720 mW at 1300 nm, corresponding to ~144 mW at the sample. The lowest power used was 640 mW, corresponding to ~128 mW at the sample. Four exposures of 300 ms were used as before, in addition to one exposure of 3 s.

### Penetration depth in fixed slices

An epifluorescence microscope was constructed above the sample stage of the previously described three-photon instrument, consisting of an objective (Zeiss 421452-9880-000), tube lens (Thor Labs AC508-150-A-ML) and camera (PCO Edge 5.5). A dichroic mirror (make and model unknown; taken from a Green Fluorescent Protein filter cube) in the beam path reflected light from a 470 nm LED (Thor Labs M470L2) onto the sample to aid in locating the sample plane.

Animal experiments were conducted in accordance with approved institutional protocols of MIT and MGH. Brain slices were obtained from a male C57BL/6J mouse; it was anesthetized and the tissues were fixed with 4% paraformaldehyde through cardiac perfusion. The fixed brain was extracted and kept in 4% paraformaldehyde, followed by embedding in low-melting agarose. It was then sliced to varying thicknesses using a vibratome (Leica VT1000S). The slices were placed in the well of a glass-bottomed dish (MatTek P50G-0-14-F) and covered with distilled water. A 4 μL drop of QDs suspended in water (525 nm emitting nanocrystals provided by QD Vision) was placed on a 30 mm diameter coverslip and allowed to dry; this coverslip was then placed on top of the brain slice with the QDs on the inside, the excess water wiped away and the coverslip sealed using clear nail varnish.

The sample was mounted on the three-photon instrument, and the upper ‘imaging’ microscope brought into focus. The lower ‘excitation’ microscope was also focussed on an unobscured region of the QDs, to ensure the two microscopes were co-aligned. A 20 lp mm^−1^ Ronchi ruling (Edmund Optics 58-777) was placed at the image plane of the excitation microscope; the pattern projected onto the sample had a period of 2.2 μm. The brain slice was then translated towards the illumination region until the fluorescence from the QDs was not observable on the excitation microscope, even with 1 s exposure and × 10 electron multiplying gain. For thinner samples, green fluorescence was observable even when the brain slice completely overlapped the excitation region; in these cases, the brain slice was translated until it filled the field of view of the camera. Finally, the excitation light was refocused to maximize signal in the imaging microscope.

For comparison with two-photon excitation, 6 W of average power was available at 800 nm, so a larger area was illuminated. Total power at the sample was 50 mW with an illuminated area of ~600 μm × 600 μm, however the increased cross-section of two-photon excitation relative to three-photon excitation resulted in similar emission intensities in the absence of scattering. Excitation was performed by routing light from the regenerative amplifier through a separate beam path described above.

Imaging was performed with a 2 s integration time on the sCMOS camera; to compensate for vibration in the imaging microscope setup (an unavoidable consequence of having to mount the microscope so far above the table surface) 16 frames were captured and the frame exhibiting the least vibration-induced blurring was taken.

### Electrophysiology

All procedures involving animals were in accordance with the US National Institutes of Health Guide for the Care and Use of Laboratory Animals and approved by the MIT Committee on Animal Care. Hippocampal neuron cultures were prepared from postnatal day 0 or 1 Swiss Webster (Taconic) mice as previously described^17, 18^ but with the following modifications: dissected hippocampal tissue was digested with 50 units of papain (Worthington Biochem) for 6–8 min, and the digestion was stopped with ovomucoid trypsin inhibitor (Worthington Biochem). Cells were plated at a density of 20,000–50,000 per glass coverslip coated with Matrigel (BD Biosciences). Neurons were seeded in 100 μL Plating Medium containing MEM (Life Technologies), glucose (33 mM, Sigma), transferrin (0.01%, Sigma), Hepes (10 mM), Glutagro (2 mM, Corning), Insulin (0.13%, Millipore), B27 supplement (2%, Gibco), heat inactivated fetal bovine serum (7.5%, Corning). After cell adhesion, additional Plating Medium was added. AraC (0.002 mM, Sigma) was added when glia density was 50–70%. Neurons were grown at 37 °C and 5% CO_2_ in a humidified atmosphere. Cultured neurons were induced at 4 days *in vitro* (DIV) with 1.0 μL of rAAV2/8-Synapsin-CoChR-GFP (titer: 3.8 × 10^12^ particles per mL) per well. AAV particles were produced by the University of North Carolina Chapel Hill Vector Core.

Coverslips supporting the prepared neuronal cultures were mounted on glass-bottomed dishes (MatTek P50G-0-14-F) at 14-20 DIV and immersed in Tyrode solution containing 125 mM NaCl, 2 mM KCl, 3 mM CaCl_2_, 1 mM MgCl_2_, 10 mM HEPES, 30 mM glucose, 0.01 mM NBQX and 0.01 mM GABAzine. The pH was 7.3. Patch clamping was performed using a micromanipulator system (Sutter Instruments MP285 micromanipulator, MPC-200 controller and Axon Instruments CV-7B headstage). Signals from the headstage were recorded using an amplifier (Molecular Devices MultiClamp 700B) and data acquisition system (Molecular Devices Digidata 1440a), controlled using pCLAMP 10 software. Exposure was controlled by the shutter, which was controlled in turn by TTL input from the data acquisition system.

Patching was performed using borosilicate glass pipettes (Warner Instruments) with an outer diameter of 1.2 mm and a wall thickness of 0.255 mm. These were pulled to a resistance of 5–10 MΩ with a P-97 Flaming/Brown micropipette puller (Sutter Instruments) and filled with a solution containing 135 mM K-gluconate, 8 mM NaCl, 0.1 mM CaCl_2_, 0.6 mM MgCl_2_, 1 mM EGTA, 10 mM HEPES, 4 mM Mg-ATP, and 0.4 mM Na-GTP, and with pH 7.3 and 290 mOsm. To ensure accurate measurements, cells were used with access resistance between 5 and 35 MΩ. Before all experiments, the holding current was adjusted such that the measured potential was between −60 and −65 mV; holding currents were within ±100 pA in all cases.

In some cases a 60 Hz signal was present in the data; this was computationally removed from the presented data by fitting a 60 Hz sine wave and subtracting the fit.

## Results and discussion

Wide-field three-photon excitation was achieved using temporal focusing, a technique in which a high-peak-power ultrafast pulse is dispersed using a grating, before being imaged onto the sample^19, 20^. This dispersion results in temporal broadening outside of the focal plane, and since multiphoton excitation is sensitive to the reciprocal of the pulse duration (raised to the power of the number of photons in the excitation process minus one^1^), efficient multiphoton excitation only occurs within a few microns of this plane.

To achieve efficient multiphoton excitation, the optimal strategy is to increase the pulse energy until the excitation spot is saturated (that is, any further increase in pulse energy results in unacceptable deterioration of the point-spread function^21^) and then to increase either the degree of parallelization (that is, increase the number of illuminated spots) or the laser repetition rate until the limit on the acceptable average power in the sample is reached. Previously, researchers have optimized the repetition rate^3^, however we argue for an increase in parallelization, using a commercially available Ti:Sapphire regenerative amplifier pumping an OPA.

Increasing parallelization has many advantages; changing the illuminated area in a temporal focusing microscope involves merely changing the size of the spot on the grating, and hence can be trivially adapted depending on the chromophore. Changing the repetition rate of a laser, on the other hand, is often difficult and the tunable range is frequently limited. The second advantage applies to chromophores with a long excited-state lifetime, or equivalent. In the case of a phosphorescent probe, for example, lifetimes of hundreds of nanoseconds are not unusual^22^. The excited-state lifetime of a QD can also be very long; up to several microseconds in some cases^23^. Other more unusual chromophores, such as opsins, have a photocycle turnover time (equivalent to the excited state lifetime for the purpose of this analysis) on the order of 10–20 ms^24^, hence increasing the repetition rate is an extremely inefficient means of optimizing the excitation. Excessive repetition rate may even contribute to reduced quantum yield and increased photobleaching, via excited-state absorption and intersystem crossing to triplet states. In contrast, by keeping the repetition rate low and increasing the degree of parallelization, these chromophores can be efficiently excited.

The final advantage is practical; obtaining a desired wavelength with high peak power at an optimal repetition rate (~1 MHz) is difficult, whereas a commercially available OPA system can tune to many different wavelengths from the ultraviolet to the mid-infrared.

### Imaging QDs

**Figure 1:**
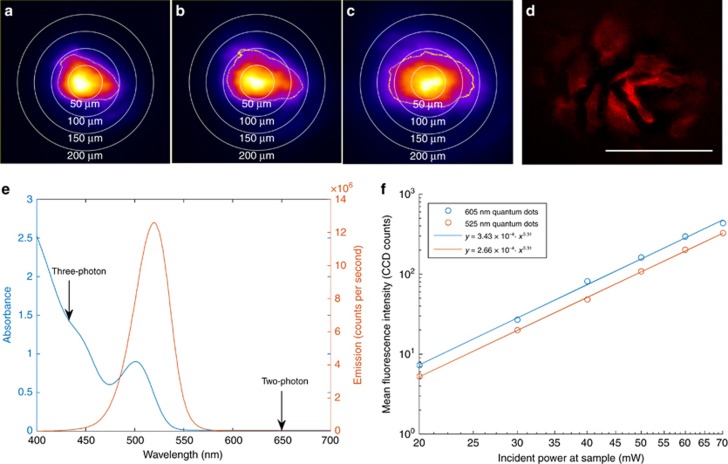
Demonstration of wide-field three-photon excitation at 1300 nm. (**a**–**c**) Effect of increasing spot size. Sample consists of a drop of quantum dots on a glass microscope slide. Color scale is arbitrary, but all images share the same scale. Circles of diameter 50, 100, 150 and 200 μm are provided as a guide to the eye, and a yellow contour indicates where the intensity drops below 25% of peak. (**a**) × 2.5 beam expander, 0.7 s exposure. (**b**) × 3.5 beam expander, 1.5 s exposure. (**c**) × 5 beam expander, 10 s exposure. (**d**) Fixed HeLa cells stained with QDot 605 quantum dots. Power at the sample is 80 mW, integration time is 10 s, the scale bar is 100 μm and flat-field correction is applied to compensate for non-uniform excitation. (**e**) Excitation and emission spectra of the 525 nm quantum dots. Arrows at 650 and 433 nm indicate the location on the one-photon absorption spectrum corresponding to two- and three-photon excitation, respectively. (**f**) Fluorescence intensity as a function of average power, demonstrating three-photon excitation.

To demonstrate efficient wide-field three-photon excitation at 1300 nm, images of a layer of 525 nm QDs were taken with several different beam expanders (Figure 1a–1c). To further validate the capability of this system for wide-field excitation, fixed HeLa cells stained with a 605 nm QD-streptavidin conjugate were imaged. A large field of view of up to ~150 μm diameter can be observed (Figure 1d). The microscope’s axial resolution was determined to be 3.0 μm median FWHM (Supplementary Fig. 2), comparable to the theoretical value for two-photon microscopy^25^ of ~1.8 μm, for a 1.0 NA lens, 1300 nm excitation and refractive index of 1.333. Compromises in axial resolution are therefore not necessary in order to employ this technique.

Figure 1e plots the absorption and emission of the 525 nm QDs. In particular, it should be noted that two-photon absorption at 1300 nm is extremely unlikely, as there are no absorption features in the one-photon spectrum near 650 nm. A similar absorption profile was obtained from the manufacturer of the 605 nm QD-streptavidin conjugates. As an additional precaution, an 850 nm long-pass filter was used to avoid one- and two-photon excitation by residual frequency-converted light from the OPA. Further confirmation of the three-photon nature of the excitation can be found in Figure 1f; the fluorescence intensity for both 525 and 605 nm QDs scales as the laser power to the power 3.31. The fact that the power scaling is not an integer indicates a contribution from four-photon excitation, consistent with the absorption cross-section of a QD which increases significantly at shorter wavelengths.

Efficient three-photon excitation can therefore be performed over a field of view sufficient to encompass several cells. Integration times of less than one second were possible with a spot size of ~100 μm diameter. Owing to the cubic dependence on excitation power, increased excitation can be achieved with modest reduction in illuminated area or increase in incident power.

### Compatibility with *in vivo* excitation

To demonstrate compatibility of three-photon wide-field excitation with biological specimens, QDs were injected into a mouse and imaged. Supplementary Videos 1 and 2 show focal stacks of vasculature in a live mouse brain. QDs were injected into the tail vein and allowed to circulate. Microvessels were subsequently imaged using the three-photon instrument through a glass window implanted in the skull. The 5 s integration time and ~250 μm diameter field of view are competitive with existing three-photon excitation methods^3^, and the 166 mW (Supplementary Video 1)/138 mW (Supplementary Video 2) incident power (with × 2.5 beam expander) caused no observable damage to the mouse.

A map of the maximum irradiance for 166 mW incident power can be seen in Supplementary Fig. 4. The aberrated mode that was caused by a misalignment in the OPA was compensated by the extra power available from the OPA; peak irradiance was ~15 W mm^−2^. After imaging, the mouse was allowed to recover from anesthesia, and when observed after an hour, no evidence of any neurological damage was present.

No attempt was made to maximize penetration depth, as tissue scattering of the emission light would limit the achievable depth to approximately the scattering mean-free path in tissue. Optimization was instead made for field-of-view and integration time. Nevertheless, for the broader use-case of three-photon excitation, an exploration of the penetration depth follows.

### Penetration through fixed brain slices

**Figure 2:**
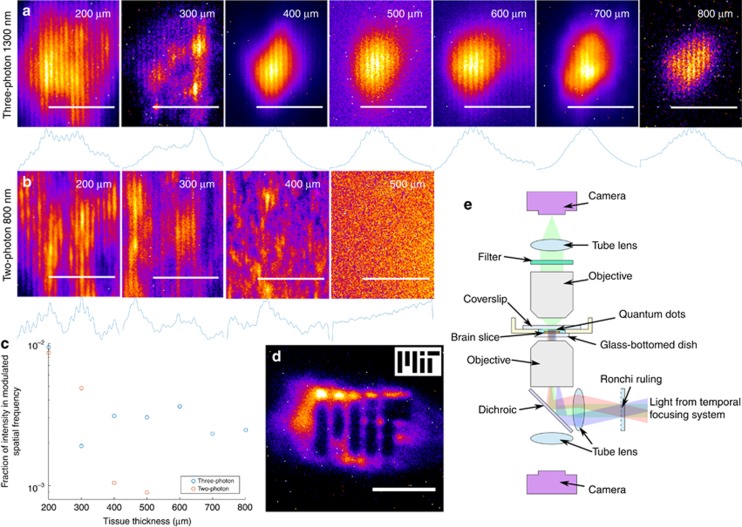
Establishing the achievable penetration depth into fixed brain tissue. (**a**) Three-photon excitation at 1300 nm through fixed tissue slices from 200 to 800 μm, with a × 2.5 beam expander. Scale bars=25 μm. A projection along the *Y* axis is shown below each image. (**b**) Two-photon excitation at 800 nm through fixed tissue slices from 200 to 500 μm. Scale bars=25 μm. A projection along the *Y* axis is shown below each image. (**c**) Summarizes the data in **a** and **b**; it plots the intensity in the modulated spatial frequency as a fraction of the intensity over the whole image. (**d**) The MIT logo projected through 700 μm of fixed brain tissue onto a layer of quantum dots using a × 3.5 beam expander. The scale bar is 25 μm, and an illustration of the mask itself is inset. A discussion of the minor image distortion observable in this figure can be found in the Supplementary Information. (**e**) The optical layout for this experiment is shown.

Since one purpose of three-photon excitation is to penetrate deeper into tissue than two-photon excitation, experiments were performed to establish the achievable penetration depth of the excitation light. A Ronchi ruling with a period of 20 line-pairs per millimeter was placed at the intermediate image plane of the microscope, such that an image of the ruling was projected onto the sample. In addition, a microscope was constructed on top of the existing one, such that the far side of the sample could be observed (Figure 2e). Fixed brain slices of varying thickness were then placed on the microscope, and a layer of QDs placed atop them, opposite the excitation objective and in the focal plane of the imaging objective. By varying the thickness of the brain slices and observing whether the modulation pattern was still present, a measure of the minimum achievable penetration depth could be determined.

The projected pattern had a period of 2.2 μm at the sample; this was deemed suitable for resolving cellular features. These images can be seen in Figure 2, along with a diagram of the experimental configuration. The experiment was repeated using two-photon excitation at 800 nm to provide a performance baseline; these results indicate that the pattern is all but unobservable by 400 μm (Figure 2b), consistent with the 250 μm penetration depth into fixed tissue achieved by Papagiakoumou *et al.*^11^. In contrast, three-photon excitation at 1300 nm achieved a penetration depth of up to 800 μm, albeit with significantly reduced excitation power. By 900 μm, the spot was unobservable, hence it was impossible to determine whether the pattern was successfully projected or not.

To confirm that arbitrary patterns could also be projected, an MIT logo was used as a photomask (Figure 2d). Careful observers will note that there is a small amount of astigmatism present in the image; this is discussed in the Supplementary Information.

Figure 2c plots the fraction of intensity in the modulated spatial frequency over the intensity in the whole image. It was calculated by subtracting the mean value of each image and taking a 2D Fourier transform; the intensity of the two peaks corresponding to the modulation frequency were summed, then divided by the sum over all frequencies. For uniform illumination with a superimposed sinusoidal pattern and infinite extent, this value tends to 1; lower values indicate a loss of contrast at the spatial frequency of interest, or alternatively, increased contributions from other spatial frequencies caused by either non-uniform excitation or fluorophore distribution. This metric is illustrated in Supplementary Fig. 5, and examples of different images and their corresponding values can be seen in Supplementary Fig. 6.

Care was taken to ensure that the occluding region of the brain tissue was located within ~100–300 μm of the cortex surface, to maximize the relevance of the data to conventional imaging. For comparison, the experiment was repeated with the focus as far below the surface of the brain as possible, and it was still possible to resolve the grating up to 500 μm into the sample, despite the clear increase in scattering of the deeper tissue (Supplementary Fig. 7). In addition, since fixation typically increases the scattering coefficient of tissue^26^, it is expected that this penetration depth represents a lower bound; live tissues will have lower scattering coefficients and larger penetration depths, as demonstrated by Begue *et al.*^27^ who managed to project two-photon temporal focusing patterns through a 550-μm-thick live brain slice, albeit using a longer wavelength (950 nm) and coarser features (on the order of 10 μm) than tested here. If the increase in performance due to the use of live tissue and coarser features applies equally for three-photon patterning as it does for two-photon patterning, the grating pattern could be preserved up to twice as far as for two-photon excitation, provided sufficient power is available.

### Optogenetic excitation

The earliest example of multiphoton excitation for optogenetics was performed by Mohanty *et al.*^28^, who focussed a single point onto a neuron to excite calcium activity in cell culture and hippocampal brain slices. Other researchers have found that, while two-photon single-point scanning can trigger an action potential in an opsin-expressing neuron^29, 30, 31^, two-photon wide-field excitation is more effective at triggering the action potential^5, 32, 33, 34^. In a report using a similar laser configuration to our high-power femtosecond OPA system, a home-built three-stage OPA system was used to illuminate an entire fly brain, without spatial selectivity, to demonstrate that two-photon excitation of opsins is not limited to small numbers of neurons—potentially the entire brain of an animal can be exposed simultaneously^35^.

**Figure 3:**
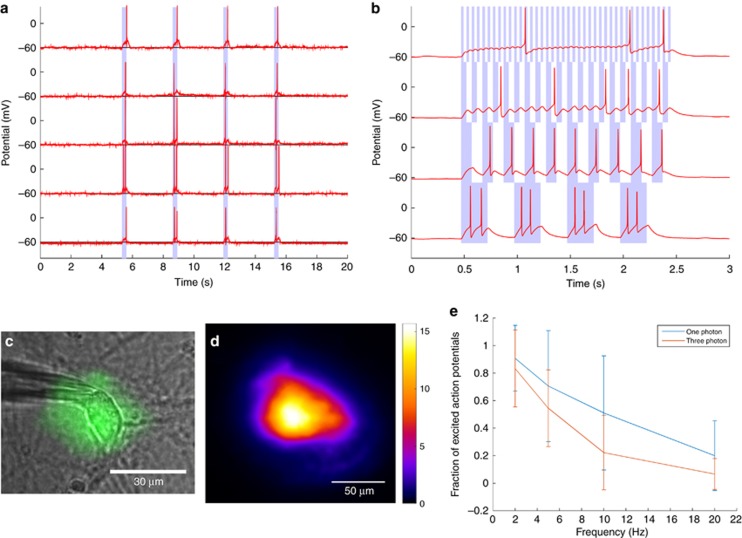
Three-photon optogenetic excitation at 1300 nm. (**a**) Representative examples of cultured neurons excited by three-photon excitation. The duration of exposure is indicated by the shaded area. (**b**) An example of the response to different excitation frequencies (2, 5, 10 and 20 Hz in ascending order). Exposure is indicated by the shaded area. (**c**) An illustration of the patched neuron with the three-photon excitation pattern superimposed. (**d**) Irradiance map of the spot, assuming 52 mW total integrated power at the sample. Units of irradiance are W mm^−2^. (**e**) Comparison of the 470 nm one-photon (*n*=19) excitation probability and the 1300 nm three-photon (*n*=9) excitation probability for cells expressing CoChR as a function of excitation frequency. Error bars indicate one standard deviation from the mean.

Demonstrating three-photon wide-field excitation of a neuron therefore serves both as an excellent test of system performance, as well as proof that wide-field three-photon excitation is possible for chromophores other than QDs. To maximize the photocurrent, a recently-developed opsin called CoChR^17^ was used. CoChR has a higher photocurrent than other opsins perhaps in part due to slow off-kinetics that enables more charge to enter the cell for a given photon dosage, as well as potentially superior membrane trafficking properties. Use of this opsin maximizes the possibility of triggering an action potential, given the low excitation efficiency of three-photon excitation versus conventional one-photon excitation. Figure 3a illustrates that it was possible to repeatedly trigger an action potential by three-photon excitation at 1300 nm in cultured neurons. Tests were also performed on non-transfected control cells, obtained from the same animal as the transfected cells (Supplementary Fig. 9). Although the power levels were occasionally high enough to trigger a damage-mediated action potential (consistent with Hirase *et al.*^36^), any damage was evident by an increase in the resting potential, and the results were not repeatable—further excitation resulted in higher and higher resting potentials and ultimately cell death. Power levels required to cause photodamage were around 64±10 mW (*n*=22 cells) at the sample, as compared with the power required to excite an action potential, which was around 51±11 mW (*n*=14 cells); errors indicate one standard deviation from the mean. This safety margin was found to be sufficient in the majority of cases to excite a cell repeatedly without causing significant damage, with damage defined as a pronounced rise in the resting potential, such that more than 100 pA hold current was required for a −60 mV voltage clamp. It is likely that this margin will be more pronounced *in vivo*, as the neurons are likely to be healthier compared with cell culture, and the presence of the vascular system can help dissipate heat in the case of thermally mediated damage mechanisms.

Results indicate that three-photon excitation of action potentials is consistently achievable (*n*=14 cells). Further investigation of the maximum excitation frequency (*n*=9 cells) illustrates the low-pass filtering response of the neurons under test; as the excitation frequency rose, the fraction of successful excitation events dropped. This is consistent with experiments performed using modulated one-photon excitation at 470 nm (see Figure 3d and Supplementary Fig. 10), as well as the mean minimum pulse duration required to excite an action potential of 52 ms, ± 20 ms standard deviation (*n*=7 cells). It is therefore safe to conclude that the inability to excite action potentials at higher frequencies is not due to the efficiency of three-photon excitation, since one-photon excitation suffers from the same limitations. Rather, it is due to either the CoChR off-kinetics, the low spontaneous spike rate of the patched neurons^37^, subtle effects due to the cell culture preparation, or any combination thereof. Indeed, the inability of our system to excite spike trains at a rate greater than ~10–20 Hz in cell culture is not unique; the maximum excitation frequencies in cell culture reported by several other authors are similar, including for Channelrhodopsin-2^5, 38^, C1V1_TT_^17^ and ReaChR^39^.

The primary damage mechanism potentially places a strong limitation on the penetration depth. If it is mediated by a single-photon absorption event, penetration will be limited as the tissue surface will be damaged as the excitation power is increased to compensate for tissue attenuation. If, however, it is mediated by multiphoton absorption, damage will be confined to the temporal focusing plane, and hence penetration depths will not be significantly affected.

Literature results suggest that damage is primarily multiphoton in nature^40, 41, 42, 43, 44^; penetration depths of well over a millimeter were achieved without observable tissue damage^3^. Nevertheless, an experiment was performed to establish the primary damage mechanism. Cells were patched and illuminated as before, but this time the microscope was defocused by ~180 μm from the cell surface, in order to maintain approximately the same photon flux but reduce multiphoton excitation to negligible levels. Even with average intensities at least double the previously determined damage threshold, no cells suffered any observable damage for either 4 × 300 ms exposures, or a subsequent 1 × 3 s exposure, whereas by translating back to the focal plane afterwards and illuminating the cell, all but two cells could be damaged (82%, *n*=11). Example plots of the membrane potential under current clamp during these experiments can be seen in Supplementary Fig. 11. From this we conclude that photodamage is likely primarily multiphoton in nature, but we should emphasize that, as with all multiphoton experiments, there may also be a very small degree of damage caused by single-photon heating.

## Conclusions

Overall, wide-field three-photon excitation overcomes many of the limitations of three-photon point-scanning, all without the need to significantly compromise axial resolution. We demonstrate excitation in a wide array of samples, both *in vivo* and *ex vivo*, with approximately double the penetration depth compared with two photon, and we successfully excite action potentials in opsin-expressing neurons. These results indicate that future moves to excitation in live brain slices or even *in vivo* experiments are well supported.

Construction of wide-field three-photon instrumentation will become much easier with the development of commercially available OPCPA designs offering several tens of Watts of power throughout the visible and near-infrared spectrum^45, 46, 47^. Excitation can therefore be performed faster, and over larger areas compared to the Ti:Sapphire-based system used here.

## Author contributions

Experiments were conceived by CJR, DP, OTB, ESB and PTCS. Instrumentation and software was constructed by CJR, with help from DP. Quantum dots from the laboratory of MGB were assembled into test samples by CJR and OTB. The mouse bearing a cranial window was obtained from the laboratory of DF and RKJ, and was prepared for imaging by OTB. For optogenetics experiments, cells were prepared by DP and transfected by KP. Patch clamping was performed by CJR and DP. The manuscript was written by CJR, with help from all other authors.

## References

[bib1] Helmchen F, Denk W. Deep tissue two-photon microscopy. Nat Methods 2005; 2: 932–940. 16299478 10.1038/nmeth818

[bib2] Jacques SL. Optical properties of biological tissues: a review. Phys Med Biol 2013; 58: R37–R61. 23666068 10.1088/0031-9155/58/11/R37

[bib3] Horton NG, Wang K, Kobat D, Clark CG, Wise FW et al. *In vivo* three-photon microscopy of subcortical structures within an intact mouse brain. Nat Photon 2013; 7: 205–209. 10.1038/nphoton.2012.336 PMC3864872 24353743

[bib4] Yu JH, Kwon SH, Petrášek Z, Park OK, Jun SW et al. High-resolution three-photon biomedical imaging using doped ZnS nanocrystals. Nat Mater 2013; 12: 359–366. 23416726 10.1038/nmat3565

[bib5] Papagiakoumou E, Anselmi F, Bègue A, de Sars V, Glückstad J et al. Scanless two-photon excitation of channelrhodopsin-2. Nat Methods 2010; 7: 848–854. 20852649 10.1038/nmeth.1505 PMC7645960

[bib6] Dodt HU, Leischner U, Schierloh A, Jährling N, Mauch CP et al. Ultramicroscopy: three-dimensional visualization of neuronal networks in the whole mouse brain. Nat Methods 2007; 4: 331–336. 17384643 10.1038/nmeth1036

[bib7] Suarez SS, Varosi SM, Dai X. Intracellular calcium increases with hyperactivation in intact, moving hamster sperm and oscillates with the flagellar beat cycle. Proc Natl Acad Sci USA 1993; 90: 4660–4664. 8506314 10.1073/pnas.90.10.4660 PMC46572

[bib8] Rowlands CJ, Wu J, Uzel SGM, Klein O, Evans CL et al. 3D-resolved targeting of photodynamic therapy using temporal focusing. Laser Phys Lett 2014; 11: 115605. 25620902 10.1088/1612-2011/11/11/115605 PMC4301304

[bib9] Rowlands CJ, Bruns OT, Bawendi MG, So PTC. Objective, comparative assessment of the penetration depth of temporal-focusing microscopy for imaging various organs. J Biomed Opt 2015; 20: 061107. 10.1117/1.JBO.20.6.061107 PMC4450320 25844509

[bib10] Sela G, Dana H, Shoham S Ultra-deep penetration of temporally-focused two-photon excitation In: Periasamy A, König K, So PTC editors. Proceedings of SPIE 8588, Multiphoton Microscopy in the Biomedical Sciences XIII 858824. San Francisco, CA, USA: SPIE; 2013.

[bib11] Papagiakoumou E, Bègue A, Leshem B, Schwartz O, Stell BM et al. Functional patterned multiphoton excitation deep inside scattering tissue. Nat Photon 2013; 7: 274–278.

[bib12] Oron D, Papagiakoumou E, Anselmi F, Emiliani V. Two-photon optogenetics. Prog Brain Res 2012; 196: 119–143. 22341324 10.1016/B978-0-444-59426-6.00007-0

[bib13] Yuan F, Salehi HA, Boucher Y, Vasthare US, Tuma RF et al. Vascular permeability and microcirculation of gliomas and mammary carcinomas transplanted in rat and mouse cranial windows. Cancer Res 1994; 54: 4564–4568. 8062241

[bib14] Jain RK, Munn LL, Fukumura D. Dissecting tumour pathophysiology using intravital microscopy. Nat Rev Cancer 2002; 2: 266–276. 12001988 10.1038/nrc778

[bib15] Dubertret B, Skourides P, Norris DJ, Noireaux V, Brivanlou AH et al. *In vivo* imaging of quantum dots encapsulated in phospholipid micelles. Science 2002; 298: 1759–1762. 12459582 10.1126/science.1077194

[bib16] Stroh M, Zimmer JP, Duda DG, Levchenko TS, Cohen KS et al. Quantum dots spectrally distinguish multiple species within the tumor milieu *in vivo*. Nat Med 2005; 11: 678–682. 15880117 10.1038/nm1247 PMC2686110

[bib17] Klapoetke NC, Murata Y, Kim SS, Pulver SR, Birdsey-Benson A et al. Independent optical excitation of distinct neural populations. Nat Methods 2014; 11: 338–346. 24509633 10.1038/nmeth.2836 PMC3943671

[bib18] Chow BY, Han X, Dobry AS, Qian XF, Chuong AS et al. High-performance genetically targetable optical neural silencing by light-driven proton pumps. Nature 2010; 463: 98–102. 20054397 10.1038/nature08652 PMC2939492

[bib19] Oron D, Tal E, Silberberg Y. Scanningless depth-resolved microscopy. Opt Express 2005; 13: 1468–1476. 19495022 10.1364/opex.13.001468

[bib20] Zhu GH, van Howe J, Durst M, Zipfel W, Xu C. Simultaneous spatial and temporal focusing of femtosecond pulses. Opt Express 2005; 13: 2153–2159. 19495103 10.1364/opex.13.002153

[bib21] Cianci GC, Wu JR, Berland KM. Saturation modified point spread functions in two-photon microscopy. Microsc Res Tech 2004; 64: 135–141. 15352084 10.1002/jemt.20071

[bib22] Choi H, Tzeranis DS, Cha JW, Clémenceau P, de Jong SJ et al. 3D-resolved fluorescence and phosphorescence lifetime imaging using temporal focusing wide-field two-photon excitation. Opt Express 2012; 20: 26219–26235. 23187477 10.1364/OE.20.026219 PMC3601594

[bib23] Moreels I, Lambert K, Smeets D, De Muynck D, Nollet T et al. Size-dependent optical properties of colloidal PbS quantum dots. ACS Nano 2009; 3: 3023–3030. 19780530 10.1021/nn900863a

[bib24] Zhang F, Vierock J, Yizhar O, Fenno LE, Tsunoda S et al. The microbial opsin family of optogenetic tools. Cell 2011; 147: 1446–1457. 22196724 10.1016/j.cell.2011.12.004 PMC4166436

[bib25] Zipfel WR, Williams RM, Webb WW. Nonlinear magic: multiphoton microscopy in the biosciences. Nat Biotechnol 2003; 21: 1369–1377. 14595365 10.1038/nbt899

[bib26] Pitzschke A, Lovisa B, Seydoux O, Haenggi M, Oertel MF et al. Optical properties of rabbit brain in the red and near-infrared: changes observed under *in vivo*, postmortem, frozen, and formalin-fixated conditions. J Biomed Opt 2015; 20: 025006. 10.1117/1.JBO.20.2.025006 25706688

[bib27] Bègue A, Papagiakoumou E, Leshem B, Conti R, Enke L et al. Two-photon excitation in scattering media by spatiotemporally shaped beams and their application in optogenetic stimulation. Biomed Opt Express 2013; 4: 2869–2879. 24409387 10.1364/BOE.4.002869 PMC3862165

[bib28] Mohanty SK, Reinscheid RK, Liu XB, Okamura N, Krasieva TB et al. In-depth activation of channelrhodopsin 2-sensitized excitable cells with high spatial resolution using two-photon excitation with a near-infrared laser microbeam. Biophys J 2008; 95: 3916–3926. 18621808 10.1529/biophysj.108.130187 PMC2553121

[bib29] Prakash R, Yizhar O, Grewe B, Ramakrishnan C, Wang N et al. Two-photon optogenetic toolbox for fast inhibition, excitation and bistable modulation. Nat Methods 2012; 9: 1171–1179. 23169303 10.1038/nmeth.2215 PMC5734860

[bib30] Packer AM, Russell LE, Dalgleish HWP, Häusser M. Simultaneous all-optical manipulation and recording of neural circuit activity with cellular resolution *in vivo*. Nat Methods 2014; 12: 140–146. 25532138 10.1038/nmeth.3217 PMC4933203

[bib31] Zhu P, Narita Y, Bundschuh ST, Fajardo O, Schärer YP et al. Optogenetic dissection of neuronal circuits in zebrafish using viral gene transfer and the tet system. Front Neural Circuits 2009; 3: 21. 20126518 10.3389/neuro.04.021.2009 PMC2805431

[bib32] Andrasfalvy BK, Zemelman BV, Tang JY, Vaziri A. Two-photon single-cell optogenetic control of neuronal activity by sculpted light. Proc Natl Acad Sci USA 2010; 107: 11981–11986. 20543137 10.1073/pnas.1006620107 PMC2900666

[bib33] Packer AM, Peterka DS, Hirtz JJ, Prakash R, Deisseroth K et al. Two-photon optogenetics of dendritic spines and neural circuits. Nat Methods 2012; 9: 1202–1205. 23142873 10.1038/nmeth.2249 PMC3518588

[bib34] Paluch-Siegler S, Mayblum T, Dana H, Brosh I, Gefen I et al. All-optical bidirectional neural interfacing using hybrid multiphoton holographic optogenetic stimulation. Neurophotonics 2015; 2: 031208. 26217673 10.1117/1.NPh.2.3.031208 PMC4512959

[bib35] Hsiao PY, Tsai CL, Chen MC, Lin YY, Yang SD et al. Non-invasive manipulation of Drosophila behavior by two-photon excited red-activatable channelrhodopsin. Biomed Opt Express 2015; 6: 4344–4352. 26601000 10.1364/BOE.6.004344 PMC4646544

[bib36] Hirase H, Nikolenko V, Goldberg JH, Yuste R. Multiphoton stimulation of neurons. J Neurobiol 2002; 51: 237–247. 11984845 10.1002/neu.10056

[bib37] Mizuseki K, Diba K, Pastalkova E, Buzsáki G. Hippocampal CA1 pyramidal cells form functionally distinct sublayers. Nat Neurosci 2011; 14: 1174–1181. 21822270 10.1038/nn.2894 PMC3164922

[bib38] Campagnola L, Wang H, Zylka MJ. Fiber-coupled light-emitting diode for localized photostimulation of neurons expressing channelrhodopsin-2. J Neurosci Methods 2008; 169: 27–33. 18187202 10.1016/j.jneumeth.2007.11.012

[bib39] Lin JY, Knutsen PM, Muller A, Kleinfeld D, Tsien RY. ReaChR: a red-shifted variant of channelrhodopsin enables deep transcranial optogenetic excitation. Nat Neurosci 2013; 16: 1499–1508. 23995068 10.1038/nn.3502 PMC3793847

[bib40] Débarre D, Olivier N, Supatto W, Beaurepaire E. Mitigating phototoxicity during multiphoton microscopy of live drosophila embryos in the 1.0-1.2 μm wavelength range. PLoS One 2014; 9: e104250. 25111506 10.1371/journal.pone.0104250 PMC4128758

[bib41] Daddysman MK, Tycon MA, Fecko CJ. Photoinduced damage resulting from fluorescence imaging of live cells. Methods Mol Biol 2014; 1148: 1–17. 24718791 10.1007/978-1-4939-0470-9_1

[bib42] Koester HJ, Baur D, Uhl R, Hell SW. Ca^2+^ fluorescence imaging with pico- and femtosecond two-photon excitation: signal and photodamage. Biophys J 1999; 77: 2226–2236. 10512842 10.1016/S0006-3495(99)77063-3 PMC1300503

[bib43] Hopt A, Neher E. Highly nonlinear photodamage in two-photon fluorescence microscopy. Biophys J 2001; 80: 2029–2036. 11259316 10.1016/S0006-3495(01)76173-5 PMC1301392

[bib44] Chu SW, Tai SP, Ho CL, Lin CH, Sun CK. High-resolution simultaneous three-photon fluorescence and third-harmonic-generation microscopy. Microsc Res Tech 2005; 66: 193–197. 15889423 10.1002/jemt.20160

[bib45] Riedel R, Stephanides A, Prandolini MJ, Gronloh B, Jungbluth B et al. Power scaling of supercontinuum seeded megahertz-repetition rate optical parametric chirped pulse amplifiers. Opt Lett 2014; 39: 1422–1424. 24690803 10.1364/OL.39.001422

[bib46] Prandolini MJ, Höppner H, Hage A, Schulz M, Tavella F et al First experimental results towards a 100 W wavelength tunable femtosecond OPCPA. In: Clarkson WA, Shori RK editors. SPIE LASE 9342, Solid State Lasers XXIV: Technology and Devices, 93421E. San Francisco, CA, USA: SPIE; 2015.

[bib47] Kraemer D, Cowan ML, Hua RZ, Franjic K, Dwayne Miller RJ. High-power femtosecond infrared laser source based on noncollinear optical parametric chirped pulse amplification. J Opt Soc Am B 2007; 24: 813–818.

