# Supplementary material for: Wide-field three-photon excitation in biological samples
Source: Light Sci Appl. 2017 May 5;6(5):e16255–. doi: 10.1038/lsa.2016.255 (PMC5687557; doi:10.1038/lsa.2016.255)
Supplement: Supplementary Information [file lsa2016255x1.pdf]

Supplementary Information for Wide-field Three-Photon Excitation in Biological Samples

Christopher J Rowlands[1], Demian Park[2], Oliver T Bruns[3], Kiryl D Piatkevich[2], Dai Fukumura[4], Rakesh K Jain[4], Moungi G Bawendi[3], Edward S Boyden[1,2,5] and Peter T C So[1,6]

[1]*Department of Biological Engineering, Massachusetts Institute of Technology, Cambridge, Massachusetts (USA)*

[2]*Media Lab, Massachusetts Institute of Technology, Cambridge, Massachusetts (USA)*

[3]*Department of Chemistry, Massachusetts Institute of Technology, Cambridge, Massachusetts (USA)*

[4]*Edwin L. Steele Laboratories, Massachusetts General Hospital and Harvard Medical School, Boston, Massachusetts (USA)*

[5]*Department of Brain and Cognitive Sciences, McGovern Institute and MIT Center for Neurobiological Engineering, Massachusetts Institute of Technology, Cambridge, Massachusetts (USA)*

[6]*Department of Mechanical Engineering, Massachusetts Institute of Technology, Cambridge, Massachusetts (USA)*

Optical diagram

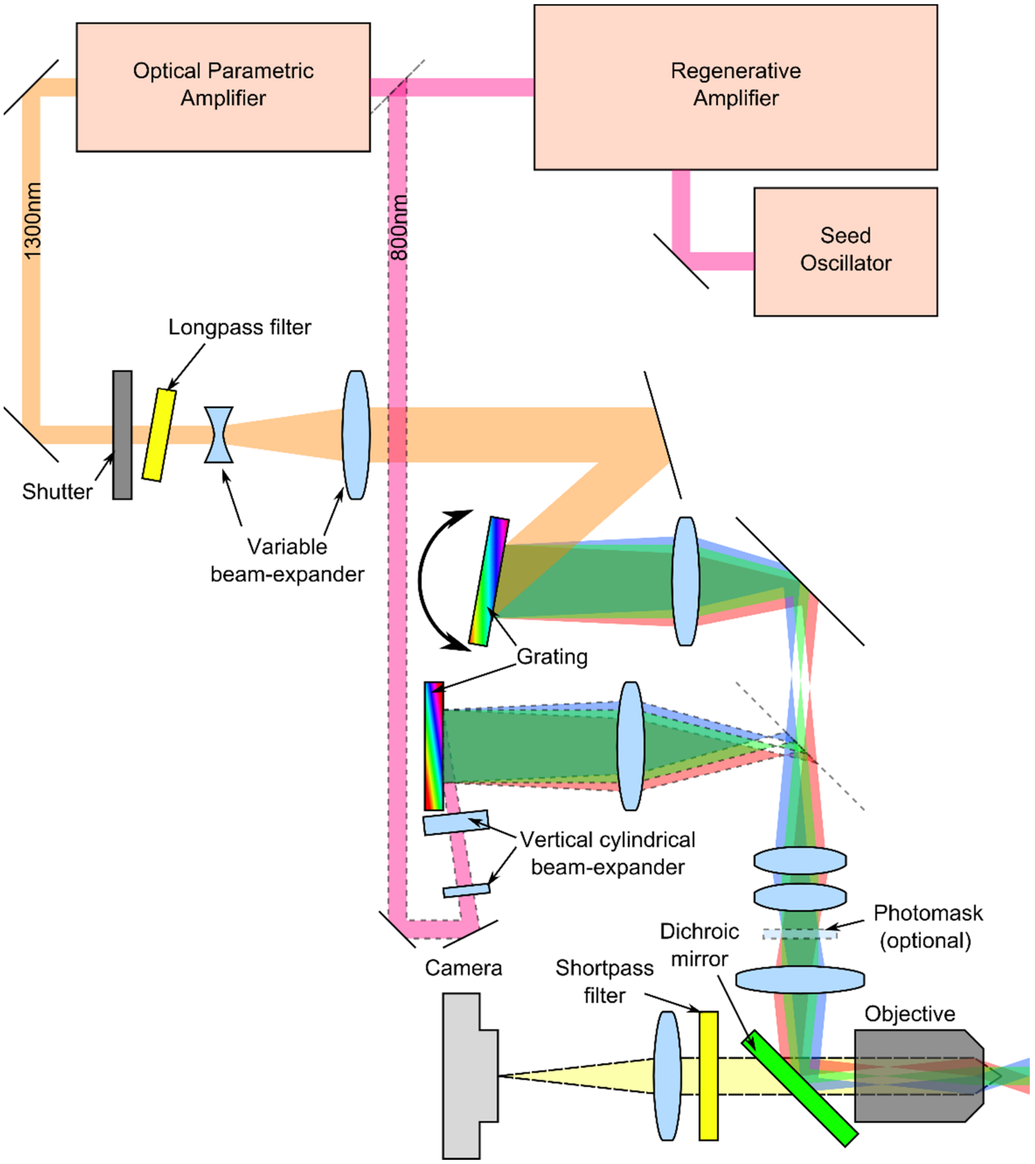

Supplementary Figure 1: Optical layout. 800 nm 35 fs pulses from the oscillator are filtered and amplified to 2.5 W and 130 fs, before being wavelength-shifted to 1300 nm, 400 mW by the Optical Parametric Amplifier (OPA). Power was controlled first by using a half waveplate and polarizer, then by moving the compressor stage in the amplifier to deliberately reduce the conversion efficiency in the OPA, or in later experiments by placing a large coverslip in the beam and rotating it. Exposure was controlled using a shutter, and a long-pass filter ensured that no unwanted harmonics of the 1300 nm beam passed into the microscope. The -1 order from the grating was imaged onto an intermediate plane just outside the microscope, before being focused onto the sample by the tube lens and objective, which was mounted on a piezo focusing drive. Emitted light passed through the dichroic; residual 1300 nm light was rejected by the short-pass filter and the fluorescence was imaged onto the camera. An optional two-photon excitation path was also available; light from the regenerative amplifier was intercepted before the OPA and routed onto a separate grating, via a cylindrical beam expander. Owing to the large amounts of power available, the whole grating surface was illuminated at an oblique angle, with the -1 order from the grating propagating down the optical axis. A removable mirror could be added to steer the resulting beam into the microscope along the path used for 1300 nm excitation.

Axial Resolution

The fitting function for the axial excitation point-spread function was taken to be the square-root of a Lorentzian function, in accordance with previous results from the literature [1]:

$$I = A\sqrt{\frac{\frac{\Gamma}{2}}{(z-z_0)^2+\left(\frac{\Gamma}{2}\right)^2}} + c$$

Where I is the measured intensity, A is a scaling term, Γ is the Lorentzian width parameter, z is the position along the z axis, z_0 is the location of the intensity maximum, and c is an offset due to a constant background intensity and / or a numerical offset on the camera. By rearrangement of the above equation, it can be shown that the full-width half-maximum of this distribution is equal to $\Gamma\sqrt{3}$.

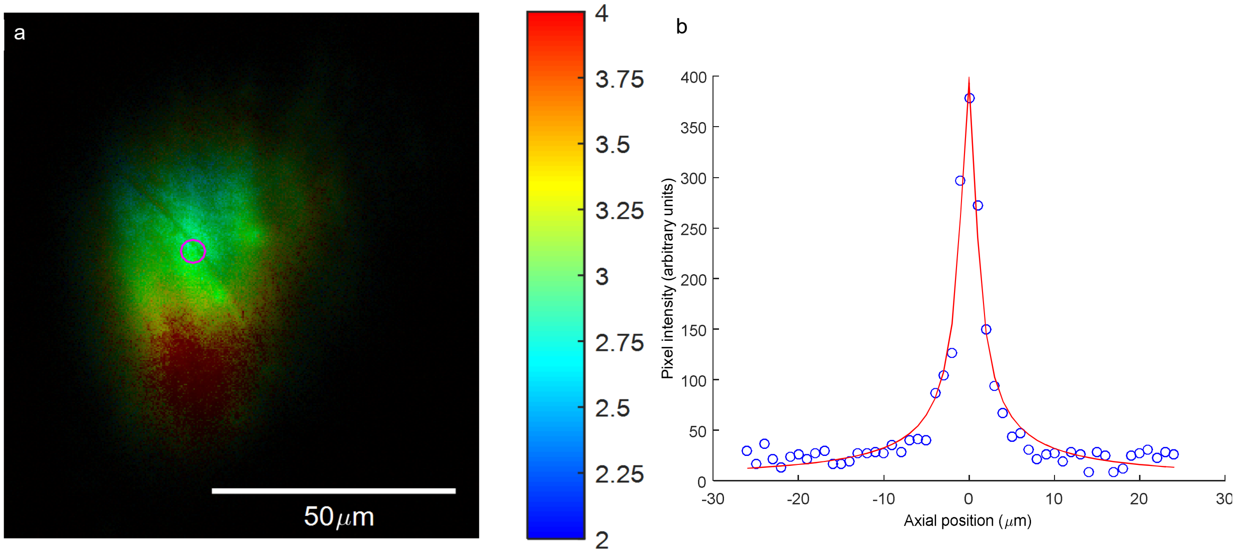

Supplementary Figure 2: (a) Measured full-width half-maximum axial resolution (in units of microns) for three-photon temporal focusing microscopy obtained by scanning the excitation plane through a thin layer of quantum dots. Hue denotes axial resolution whereas intensity corresponds to the intensity of the sample. (b) Example Z-scan for the pixel highlighted with a magenta circle in (a).

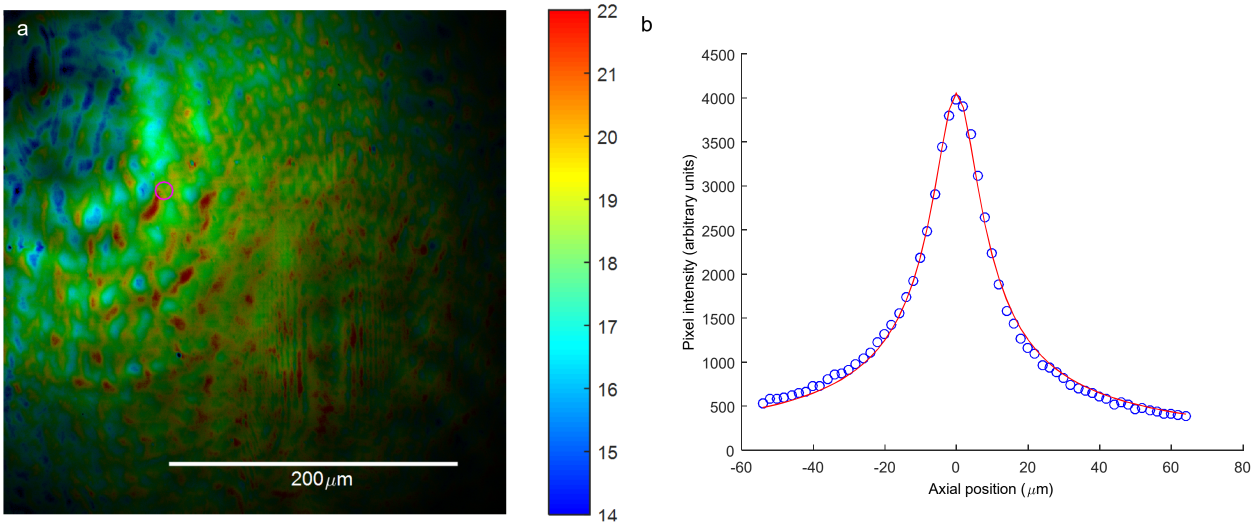

Supplementary Figure 3: (a) Measured full-width half-maximum axial resolution (in units of microns) for two-photon temporal focusing microscopy obtained by scanning the excitation plane through a thin layer of quantum dots. Hue denotes axial resolution whereas intensity corresponds to the intensity of the sample. (b) Example Z-scan for the pixel highlighted with a magenta circle in (a).

In vivo imaging

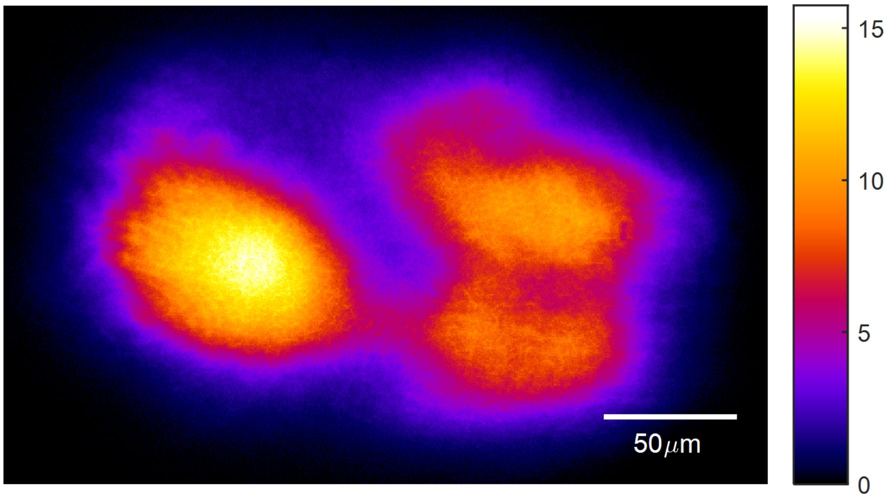

Supplementary Figure 4: Irradiance used for in vivo imaging of mouse vasculature, assuming 166 mW total integrated power at the sample. Units of irradiance are W/mm².

Correlating visual appearance and fractional in-band power

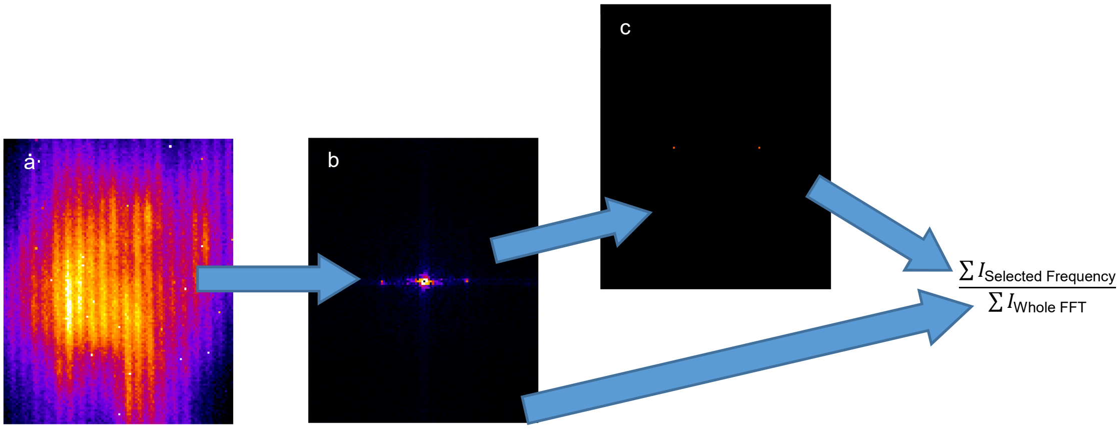

Supplementary Figure 5: Visual illustration of how the Fractional In-Band Power (FIBP) metric is calculated. The image (a) is processed to determine the 2D Fourier Transform magnitude (b). The location of the two peaks either side of the center is pre-calculated based on the known period of the sinusoidal pattern; these peaks are masked out to form (c). The sum of (c) forms the nominator, and the sum of (b) forms the denominator of the FIBP metric.

**Image**	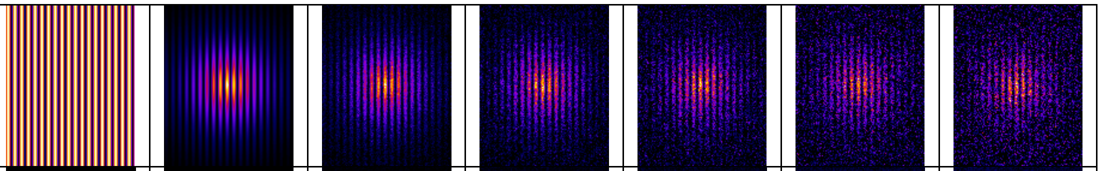						
**FFT**	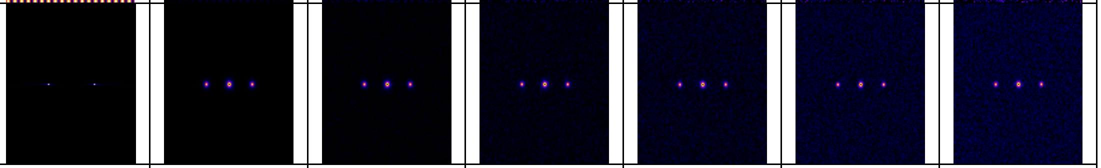						
**Photons**	Infinite	Infinite	100	33	20	14	11
**FIBP**	0.4955	0.0656	0.0168	0.0096	0.0070	0.0053	0.0044

Supplementary Figure 6: Illustration of some images, their corresponding 2D Fourier transforms, the mean number of photons in the image maxima (i.e. where the noiseless image would have a value of 1), and the resulting Fractional In-Band Power (FIBP) metric.

Penetration depth experiments

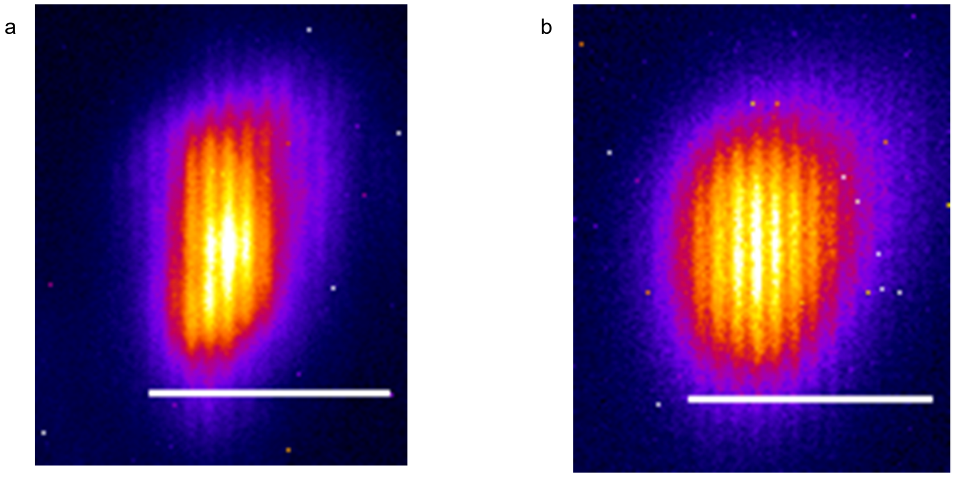

Supplementary Figure 7: Achievable penetration depths into sub-cortical scattering tissue; the focal point is located approximately 1 mm within the sample. (a) 400 µm slice thickness (b) 500 µm slice thickness.

Image distortion

Careful examination of Figure 2d will show that the vertical lines appear thinner than the horizontal lines, a phenomenon which is most pronounced on the 'T', and which occurs despite the fact that the width of both lines should be equal. After eliminating many possible explanations for this phenomenon, it appears the most likely explanation is astigmatism due to curvature of the dichroic mirror.

Evidence for this comes from direct measurement of the curvature of the dichroic, as well as the observation that the image can be brought to two different foci; one for horizontal features, and one for vertical features (see Supplementary Figure 8). To measure the flatness, the beam from a 532 nm laser diode was expanded by 5× using a beam expander (Thorlabs, BE02-05-A) and imaged using a Shack-Hartmann wavefront sensor. The beam expander focus was tuned to yield minimum wavefront curvature (as measured by Zernike mode Z_2^0) to obtain a reference measurement. The pupil diameter on the sensor was 3.57 mm, with a mean fit error of 2.7×10^{-14} arcminutes. The dichroic was then placed in the beam and the wavefront sensor moved to image the reflected beam. The fit error was unchanged, but the Zernike modes responsible for defocus (Z_2^0) and vertical astigmatism (Z_2^2) were over an order of magnitude larger than for the reference measurement. Further evidence can be found from attempts to move the mask to the optimum focal point; a layer of quantum dots was used without any intermediate scattering layer, and the MIT logo projected onto it. By moving the photomask, it was possible to locate a point where the vertical lines were in focus, and a point where the horizontal lines were in focus: a hallmark of astigmatism.

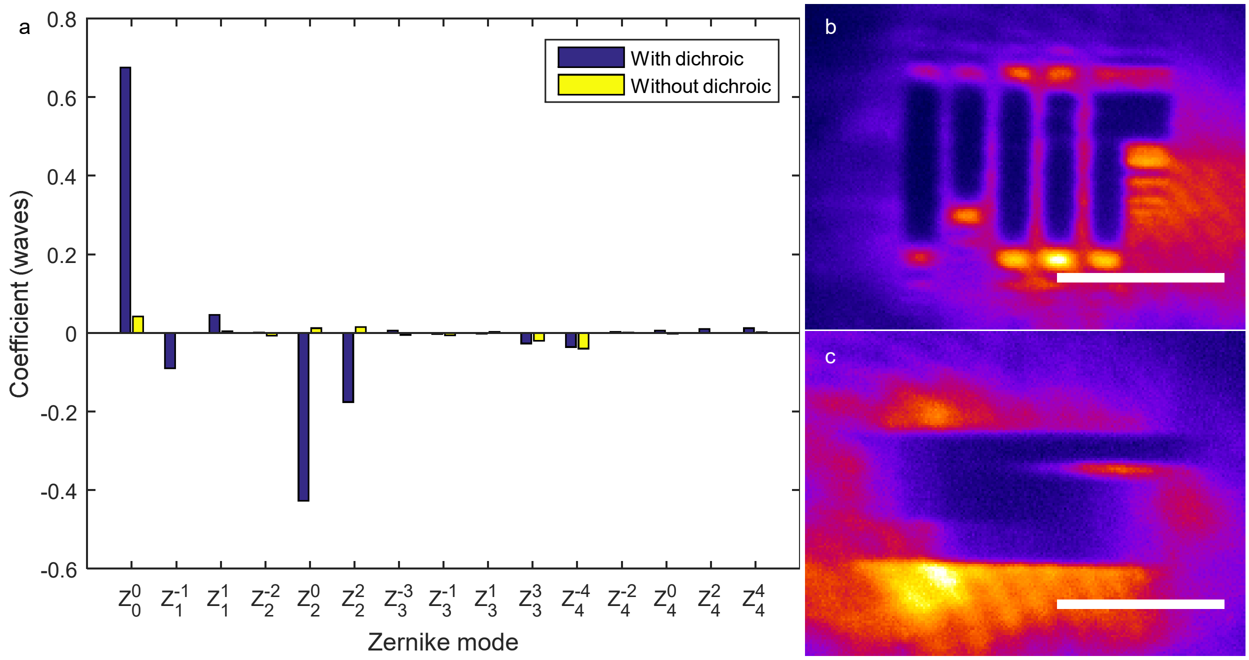

Supplementary Figure 8: Investigating astigmatism in projected images. (a) Wavefront aberration due to the dichroic, measured at 532 nm. Pupil size is 3.57 mm. (b) Projected image at the sample, with the photomask placed at the location which produced the best focus of vertical lines. Scale bar is 25 µm. (c) Projected image at the sample, with the photomask placed at the location which produced the best focus of horizontal lines. Scale bar is 25 µm.

Issues with the flatness of dichroic mirrors are known to manufacturers. The deposition of a thin film on one side of a substrate can introduce stress; the effect is to introduce curvature into the dichroic, with a more pronounced curvature along the long axis. This leads to a defocusing of the incident light (which is often unnoticed as users will compensate by moving the objective) as well as astigmatism along the long axis of the dichroic mirror. Semrock offers a range of 'imaging flat' dichroic mirrors with carefully-chosen film thicknesses and composition that can minimize this aberration, and many manufacturers will utilize a thicker substrate to achieve the same goal. Nevertheless, Edmund Optics does not offer a low-curvature model of the 86-699 1200 nm short-pass dichroic used in this paper, so we recommend that users who wish to project patterns onto the substrate by temporal focusing should custom-manufacture their dichroic mirrors.

Optogenetics control experiments

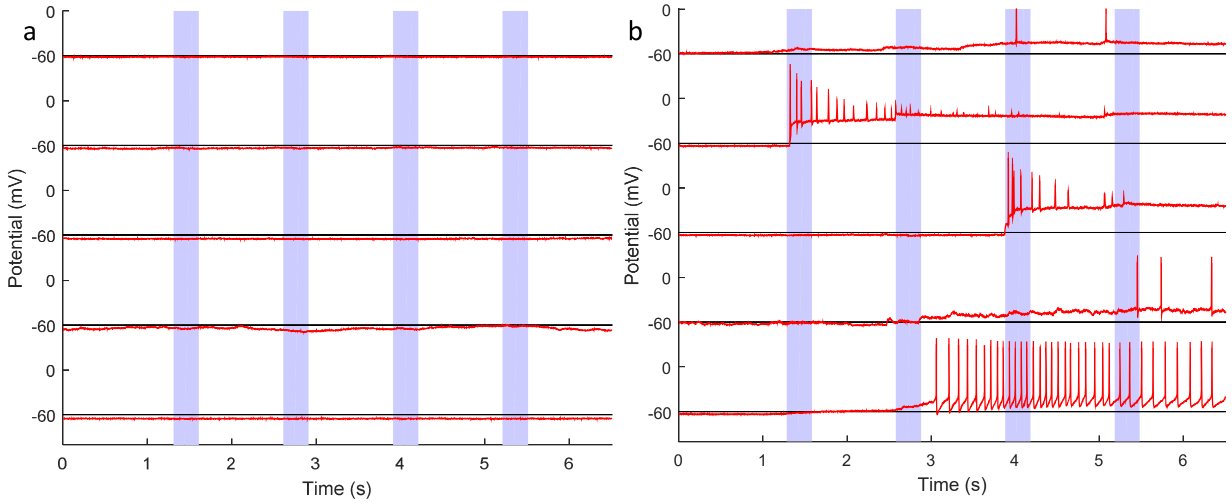

Supplementary Figure 9: example plots of control experiments on cells that were not expressing an opsin. (a) Cells which did not noticeably respond to excitation. (b) Cells which became damaged during excitation. The coloured regions indicate the duration of the light exposure.

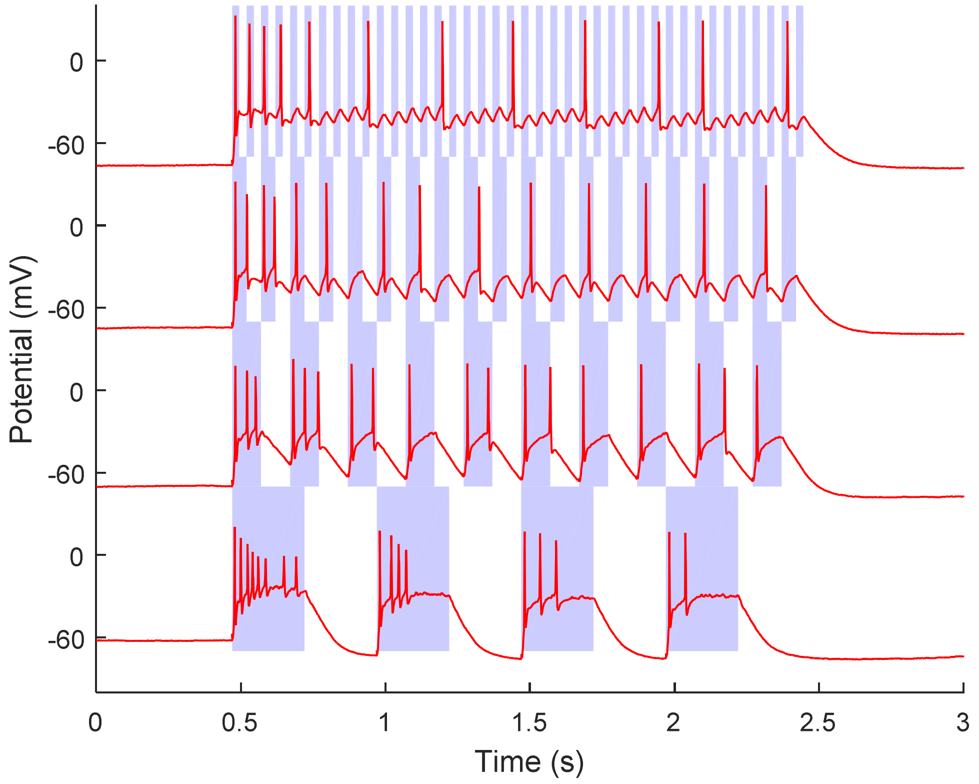

Supplementary Figure 10: Example one-photon response of CoChR to various excitation frequencies (2 Hz, 5 Hz, 10 Hz and 20 Hz from bottom to top). The exposure duration is indicated by the coloured regions.

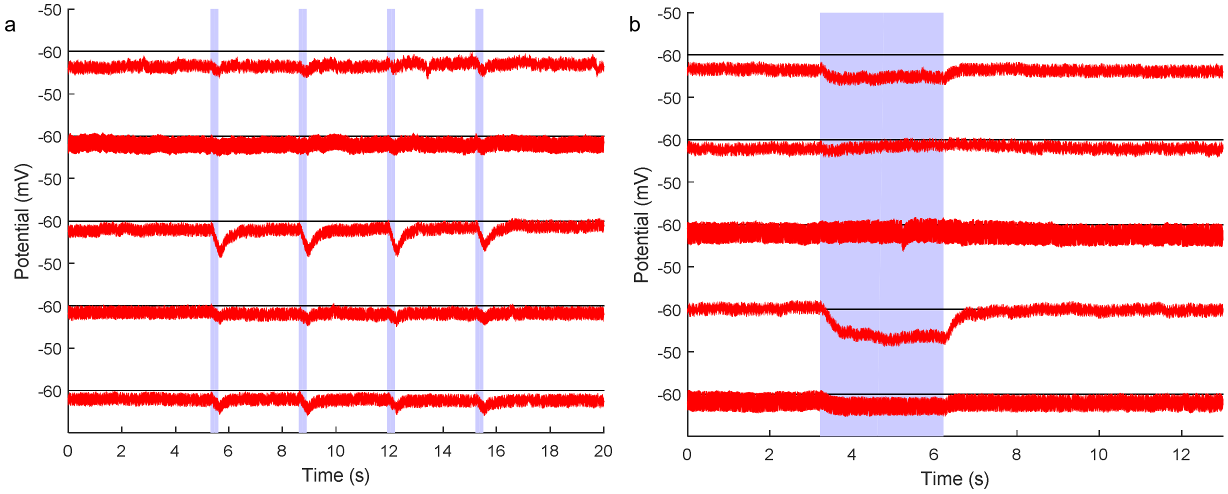

Supplementary Figure 11: Establishing the nature of the damage mechanism; blue area indicates the exposure duration. In (a), the focal plane is approximately 180 µm away from the cell surface and the power is increased more than 50% over that used in previous experiments, but the illumination conditions are otherwise identical to that in Figure 3(a). In (b) illumination durations were increased to 3 s, to ensure that damage was avoided for even the longest exposure durations. Note the different Y scale compared to Figure 3 and Supplementary Figure 8 and 9. Careful observers will note that a small but repeatable hyperpolarization of a few millivolts could be observed in response to the out-of-focus 1300 nm excitation; while a thorough investigation of this phenomenon would be beyond the scope of this paper, the phenomenon itself defies easy explanation, and further investigation is ongoing.

References

1. Dana, H. & Shoham, S. Numerical evaluation of temporal focusing characteristics in transparent and scattering media. *Opt. Express* **19,** 4937–48 (2011).
